# Football-Ear

**Published:** 1889-02-02

**Authors:** 


### FOOTBALL EAR.

"FOOTBALL" ear is by no means uncommon among those who play the "Rugby" game. It consists of an effusion of blood into the auricle, the upper and prominent part of the ear. Many observers have noticed the circumstance in football players—among the rest, the late James Hinton. Some surgeons have considered it to be due to hard blows on the ear with the ball, received in the course of the game. The real cause, however, seems to be the "scrimmage," into which some boys and men enter with such fierce determination. During this tussle the "forward" players often have the head bent down and to one side, and the arms of the opposed "forward" players press with great force upon that part of the ear which is most prominent. At the same time the desperate struggling of contending sides causes not seldom a decided congestion of the vessels of the head and neck. Granted these two conditions—a marked congestion of the head and neck, which, of course, spreads to the ears, and great pressure upon a prominent part like the auricle, it is easily seen that blood may be extravasated in considerable quantities. The injury has become so common among those who play the "Rugby" game that many "forward" players now wear tightly-fitting caps, with lateral flaps, for the purpose of protecting the ears. In many cases the injury is slight, and the effects pass away in a few days. Not seldom, however, the extravasated blood, passing through the crushed and ruptured blood-vessels and mingling with the products of exudation, gives rise to a mass of fibroid tissue, causing a true hæmatoma or blood tumour. This, in course of time, undergoes contraction, and gives to the ear a peculiarly shrivelled-up appearance, which is very unsightly. A case of this kind was reported not very long ago. The patient was a strong and healthy young fellow, twenty years of age. About six months before his appearance in the surgeon's consulting-room he had played "forwards" in a keenly-contested game, in which the "scrimmages" had been very determined. The same evening he noticed that the upper part of his left ear was much swollen in front, and that the swelling was of a dark blue colour. A medical man, to whom the swelling was shown, opened it, and let out a small quantity of fluid blood. During the following month the young man played again several times, and each time the ear was swollen as on the first occasion. At last inflammation was set up, and pus was formed. A second time an opening was made, and the pus was discharged. Afterwards the inflamed parts shrank, and became contracted until they assumed the unsightly appearance of a shrunken and diseased ear. The surgeon, to whom application was made, cut through the skin and dissected out an irregular mass of dense fibroid tissue. The wound healed rapidly, and the ear. although not restored to its natural shape and appearance, was very much improved. That cases of this kind are decidedly common among "forward" players is shown by the fact that this particular patient told the surgeon he knew of six others in his own or neighbouring clubs who had suffered in the same way as he had. A similar experience is said to be not uncommon among Cumberland wrestlers, whose attitude in wrestling is familiar to north countrymen. Pugilists also, who have had conferred upon them the posthumous honour of statues, are said to frequently show the same kind of deformed ear.

